# Homeostasis of chosen bioelements in organs of rats receiving lithium and/or selenium

**DOI:** 10.1007/s10534-016-9958-9

**Published:** 2016-07-30

**Authors:** Małgorzata Kiełczykowska, Irena Musik, Renata Żelazowska, Anna Lewandowska, Jacek Kurzepa, Joanna Kocot

**Affiliations:** Chair and Department of Medical Chemistry, Medical University of Lublin, Chodźki 4a, 20-093 Lublin, Poland

**Keywords:** Selenium, Lithium, Magnesium, Calcium, Silicon, Male rats

## Abstract

Lithium is an essential trace element, widely used in medicine and its application is often long-term. Despite beneficial effects, its administration can lead to severe side effects including hyperparathyroidism, renal and thyroid disorders. The aim of the current study was to evaluate the influence of lithium and/or selenium treatment on magnesium, calcium and silicon levels in rats’ organs as well as the possibility of using selenium as an adjuvant in lithium therapy. The study was performed on rats divided into four groups (six animals each): control-treated with saline; Li-treated with Li_2_CO_3_ (2.7 mg Li/kg b.w.); Se-treated with Na_2_SeO_3_·H_2_O (0.5 mg Se/kg b.w.); Se + Li-treated simultaneously with Li_2_CO_3_ and Na_2_SeO_3_·H_2_O (2.7 mg Li/kg b.w. and of 0.5 mg Se/kg b.w., respectively). The administration was performed in form of water solutions by stomach tube once a day for 3 weeks. In the organs (liver, kidney, brain, spleen, heart, lung and femoral muscle) the concentrations of magnesium, calcium and silicon were determined. Magnesium was increased in liver of Se and Se + Li given rats. Lithium decreased tissue Ca and co-administration of selenium reversed this effect. Silicon was not affected by any treatment. The beneficial effect of selenium on disturbances of calcium homeostasis let suggest that further research on selenium application as an adjuvant in lithium therapy is worth being performed.

## Introduction

Lithium, an essential trace element, is widely used in different fields of medicine. For many years it has been used mostly in psychiatry (Shalbuyeva et al. 2007; Młyniec et al. 2014). However, studies have revealed the possibility of its application in other cases e.g.: in therapy of thyroid diseases, neurodegenerative disorders or preventing nephrolithiasis (Camins et al. 2009; Zhang et al. 2009; Wallace 2014; Chouhan et al. 2016). Lithium chloride has been suggested to be effective in leukemia therapy (Li et al. 2015). The application of lithium is connected with considerable problems since lithium shows beneficial action only within a strongly determined range (Młyniec et al. 2014). The exceeding of the safe threshold may be accompanied with severe side effects including hyperparathyroidism, renal and thyroid disorders (McKnight et al. 2012; Lehmann and Lee 2013; Albert et al. 2013; Shine et al. 2015).

Relationships between lithium intake and bioelements homeostasis have been reported (Shalbuyeva et al. 2007; Oliveira et al. 2014; Shine et al. 2015; Harari et al. 2016). It has been suggested that the neuroprotective effects of lithium may be connected with its effect on intracellular Ca^2+^ concentrations (Wallace 2014). Hypercalcaemia is connected with lithium administration, however, this issue has been reported not to be fully investigated yet (Albert et al. 2013). Mg^2+^ and Li^+^ ions have been found to compete for binding-site of some enzymes (Dudev and Lim 2011). Our previous studies have revealed the effect of lithium treatment on silicon level in animal organs (Kiełczykowska et al. 2008).

The searching for protective agents against lithium action seems to be worth studying considering that its application in psychiatric patients is commonly long-term and irrespective of numerous side effects it is still recommended as a first-line maintenance treatment for bipolar disorder (Albert et al. 2013). Furthermore, the scientists have recently pointed that growing use of lithium may result in increased environmental contamination with this element (Tkatcheva et al. 2015). The risk for consumers resulting from the possible occurrence of lithium in drinking water is also suggested to be considered (Harari et al. 2016). As different bioelements showed protective properties against different harmful factors, we undertook the current study aiming at evaluation of the possible beneficial influence of selenium on calcium, magnesium and silicon in organs of rats exposed to lithium. Selenium—an important trace element—has already been studied in regard to its protective action against numerous factors, both chemical and physical, and the obtained results have been highly encouraging (Ghodbane et al. 2011; Hassanin et al. 2013; Jebur et al. 2014; Shen et al. 2016). An additional reason for this study was the fact that an organoselenium compound ebselen has been reported to show some lithium-mimetic properties what has made the authors suggest the possibility of future application of ebselen in bipolar disorder therapy (Masaki et al. 2016). An acknowledged inorganic supplement sodium selenite, still used in clinical practice (Savory et al. 2012; Manzanares et al. 2015) and as a supplement of animal food (Pavlović et al. 2010; Cun et al. 2015) was chosen, on account of its bioavailability.

The aim of the current study was to evaluate the influence of lithium treatment on magnesium, calcium and silicon levels in rats’ organs as well as the effectiveness of selenium as an adjuvant in lithium therapy.

## Materials and methods

### Animals

The experiment was performed on adolescent male Wistar rats (24 animals, 130–160 g body weight). The animals had free access to standard feed and drinking water. The study was carried out according to statutory bioethical standards and approved by I Local Ethical Commission of Medical University of Lublin, acceptance no. 1/2013.

### Experimental design

After 3-day-acclimatization period the rats were divided randomly into four groups (six animals each): group I (control)—treated with saline; group II (Li)—treated with lithium (as Li_2_CO_3_) at a dose of 2.7 mg Li/kg b.w.; group III (Se)—treated with selenium (as Na_2_SeO_3_·H_2_O) at a dose of 0.5 mg Se/kg b.w.; group IV (Se + Li)—treated simultaneously with lithium (Li_2_CO_3_) and selenium (Na_2_SeO_3_·H_2_O) at a dose of 2.7 mg Li/kg b.w. and of 0.5 mg Se/kg b.w., respectively. The administration was performed in form of water solutions by stomach tube. The treatment was performed once a day, for a period of 3 weeks. Basing on the body mass of each animal, measured every day before administration, the appropriate amount of selenium and/or lithium solutions was calculated. In the end of the experiment the animals were sacrificed under thiopental narcosis and samples of tissues of liver, kidney, brain, spleen, heart, femoral muscle and lung were collected. Ten % (w/v) tissue homogenates were prepared in 0.1 mol dm^−3^ Tris–HCl buffer, pH 7.4. Supernatants were obtained by centrifugation at 5000×*g* for 30 min.

### Biochemical investigations

In the prepared tissue supernatants the concentrations of magnesium, calcium and silicon were determined. Magnesium and calcium concentrations were assayed by colorimetric methods using diagnostic kits Liquick Cor-MG 60 and Liquick Cor-CALCIUM 120, respectively. The concentration of silicon was measured using the spectrophotometric method (Wielkoszyński 2000). The obtained values of the determined elements’ concentrations were expressed in μmol g^−1^ of wet tissue. The assays were performed using spectrophotometer SPECORD M40 (Zeiss Jena).

### Statistics

All statistical analyses were performed using STATISTICA program (version 10.0) The normality of data distribution was verified using Shapiro–Wilk test. The differences among the studied groups were analyzed using a one-way analysis of variance (ANOVA), followed by Tukey test (for normally distributed variables) or Kruskal–Wallis one way analysis of variance (for non-normally distributed variables). Values were considered significant with p < 0.05.

## Results

**Fig. 1:**
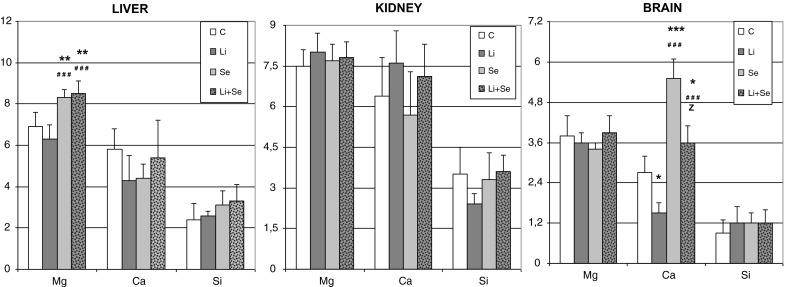
Magnesium, calcium and silicon concentrations (μmol g^−1^ of wet tissue) in organs of rats receiving lithium and/or selenium. *p < 0.05 versus control; **p < 0.01 versus control; ***p < 0.001 versus control; ^###^p < 0.001 Li-group; ^Z^p < 0.001 versus Se-group

In liver magnesium was significantly increased in animals treated by Se alone and Li + Se versus both control and Li-alone groups. The other two elements showed no distinct differences among the studied groups. In kidney no influence of any treatment on the studied bioelements was observed. In brain Mg and Si were not affected by Li and/or Se treatment. In contrast, in case of calcium Li-treatment caused a significant decrease versus control. In Se alone given rats a well-marked Ca enhancement versus both control and Li-treatment was observed. Co-administration of Li and Se resulted in a significant increase versus both control and Li-treated groups, whereas compared to Se alone group a significant depletion was noted. All the presented above results were shown on Fig. 1.

**Fig. 2:**
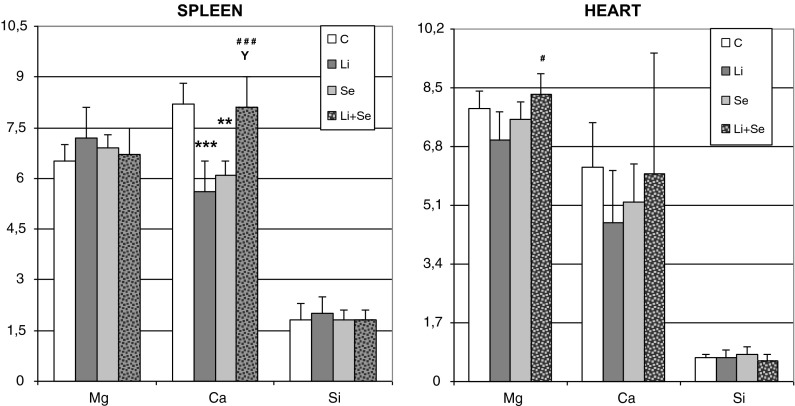
Magnesium, calcium and silicon concentrations (μmol g^−1^ of wet tissue) in organs of rats receiving lithium and/or selenium. **p < 0.01 versus control; ***p < 0.001 versus control; ^#^p < 0.05 Li-group; ^###^p < 0.001 Li-group; ^Y^p < 0.01 versus Se-group

Similarly as in brain, in spleen calcium was the only element whose homeostasis was affected. Li alone and Se alone significantly decreased its tissue concentration versus control. Co-administration of Se resulted in full restoration of Ca as in Li + Se group a well-marked increase versus Li- and Se-treatments concomitant with no difference compared to control was obtained. In heart homeostasis of the studied bioelements generally showed no disturbances caused by the used treatments, except for an Mg increase versus Li-group in Li + Se-given rats. All the presented above results were shown on Fig. 2.

**Fig. 3:**
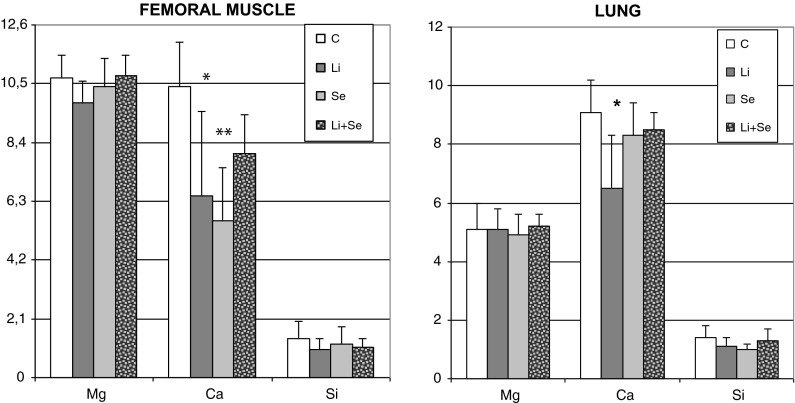
Magnesium, calcium and silicon concentrations (μmol g^−1^ of wet tissue) in organs of rats receiving lithium and/or selenium. *p < 0.05 versus control; **p < 0.01 versus control

In femoral muscle and lung tissues calcium was also the only element, influenced by any of the used treatments. In femoral muscle Se alone and Li alone caused a well-marked decrease versus control, whereas in case of Li + Se-group the insignificant depletion was observed. In lung only Li alone administration resulted in a significant Ca decrease versus control. All the presented above results were shown on Fig. 3.

## Discussion

In the current study lithium did not affect magnesium in any studied organ. Selenium alone or co-administered with Li also changed Mg level in no studied organ, except for liver, where significant increase was observed. This effect could be beneficial in patients receiving different drugs, considering the detoxifying role of liver and the evidence of relationships between magnesium and selenium level and incidence of liver disturbances (Markiewicz-Górka et al. 2011; Nangliya et al. 2015). Our previous studies revealed that intraperitoneal selenium can increase magnesium in liver and this effect was found to show a distinct dependence on the used form as a selenoorganic compound enhanced liver Mg much more considerably compared to inorganic selenite (Musik et al. 2010). In contrast Sivrikaya et al. (2013) observed no effect of intraperitoneally given sodium selenite on liver Mg of rats. No effect of lithium on magnesium, observed in the present work, is also partially consistent with one of the previous experiments performed in our department where the similar Li doses given in drinking water caused no changes or increase in Mg in rats’ organs (Kiełczykowska et al. 2007). LiCl provided with drinking water also resulted in no changes of magnesium in brain, liver, skeletal muscle and cardiac muscle of rats (Csutora et al. 2006). The lack of connections between these two elements was also found by Baltaci et al. (2014) who observed that enhancement of Li in bone of ovariectomized rats fed Zn-deficient diet was accompanied with no changes of bone magnesium. The lack of disturbances of Mg level in brain by any treatment used in the present experiment seems to be very important, given that magnesium is regarded as one of the most important elements for the central nervous system (Ghasemi et al. 2010). Magnesium is considered as a antagonist of *N*-methyl-d-aspartate (NMDA) receptors and in Mg deficiency these receptors are hyperexcitable. Despite NMDA receptors are involved in several physiologic functions as excitatory synaptic transmission, neuronal plasticity and memory, their overstimulation leads to excitotoxicity resulting in neurons’ death (de Baaij et al. 2015). Low serum Mg levels are associated with several neurological diseases such as migraine, depression, epilepsy and Alzheimer disease (AD) (Gupta et al. 1994; Gonulu et al. 2015; Gröber et al. 2015; Yary et al. 2016). In addition, the time-dependence of lithium influence on brain magnesium was observed in fish exposed to Li. While no changes were observed at the beginning, after 72 h a decrease versus control was observed (Tkatcheva et al. 2015).

In the current study calcium was proved to be the most influenced element. It seems to be consistent with the hypothesis that lithium-induced hyperparathyroidism and hypercalcaemia can be connected with decrease in the intracellular calcium uptake (Kandil et al. 2011; Albert et al. 2013). The negative relationships between calcium and lithium were reported. In ovariectomized rats fed zinc-deficient diet markedly enhanced lithium in bone was accompanied with a significant calcium decrease (Baltaci et al. 2014). A case report study revealed that in a lithium-treated patient a decrease in dentin Ca was found (Eduardo et al. 2013). In a study performed on fish an interesting, time-dependent effect of lithium on brain calcium was found. No changes were observed at the beginning, after 24 h a decrease versus control was observed, after 72 h increase, and in the end (after 96 h) no changes were observed again (Tkatcheva et al. 2015). The cytotoxicity of calcium has been recognized for about 35 years ago. A few theories explain the mechanism of excitotoxicity of Ca, and all of them emphasize the higher than given threshold cytoplasmatic calcium level as an essential factor which enters the cell into the apoptotic pathway (Szydlowska et al. 2010). The elevated Ca level within the brain tissue in animals treated with Se can suggest the higher potential for excitotoxicity development, however, this observation needs further studies on cellular level.

Impairment of calcium homeostasis was also found in pregnant women exposed to Li in their drinking water and this effect was connected with vitamin D level disturbances (Harari et al. 2016). It is consistent with the fact that renal toxicity belongs to the main lithium side effects (Harrison et al. 2016). Other scientists also reported the disturbances of vitamin D in lithium-treated patients (van Melick et al. 2014).

No significant influence of lithium and/or selenium on silicon was found in the current study. Considering lithium’s application in psychiatry and neurology it seems to be important as silicon is regarded as an essential element for brain (Santos-López et al. 2016). Previous animal study revealed the regional variations in silicon content within the brain which were independent of dietary silicon supplementation. However, supplementation of aluminum, which is potential factor for AD development, decreased the silicon content in selected brain regions (Carlise et al. 1987). In available literature data there is not too much information about relationships among these bioelements. One of our previous studies revealed that comparable doses of lithium, provided with drinking water, caused mostly no changes of Si in rats’ organs. A slightly higher dose resulted in Si increase in liver and femoral muscle, whereas a lower one decrease in kidney. A wide spectrum of Li doses, studied in that experiment, did not alter brain silicon level (Kiełczykowska et al. 2008). The connections between selenium and silicon have been poorly investigated to date. However, it was suggested that a silicon influx transporter OsNIP2;1 is the first transporter of inorganic selenium (selenite form) in plants, microorganisms and animals (Zhao et al. 2010).
